# Multiple Breath-hold Volumetric Modulated Arc Therapy Under Fluoroscopic Image Guidance with an Implanted Fiducial Marker: An Advanced Technique

**DOI:** 10.7759/cureus.2499

**Published:** 2018-04-18

**Authors:** Tsuyoshi Takanaka, Yoko Taima, Yasushi Horichi, Yasuhiro Kawamori, Koji Nobata, Kiyohide Kitagawa, Azusa Norishima, Kento Koshikawa, Tatsuya Mito, Satoshi Yoshida, Tetsuya Kawanaka, Katsuaki Matsutani, Masahiro Kawahara

**Affiliations:** 1 Department of Radiation Oncology, Kouseiren Takaoka Hospital; 2 Department of Radiation Oncology, Kanazawa University Hospital; 3 Department of Radiology, Kouseiren Takaoka Hospital; 4 Imaging Diagnosis, Kouseiren Takaoka Hospital

**Keywords:** breath-hold, vmat, fluoroscopy, fiducial markers, respiratory motion management

## Abstract

An advanced technique for multiple breath-hold volumetric modulated arc therapy (VMAT) has been proposed under fluoroscopic image guidance with a fiducial marker implanted close to a tumor. The marker coordinates on a digitally reconstructed radiography image at a gantry start angle, under a planned breath-hold condition, were transferred to the fluoroscopic image window. Then, a reference lateral line passing through the planned breath-hold marker position was drawn on the fluoroscopic image. Additional lateral lines were further added on both sides of the reference line with a distance of 3 mm as a tolerance limit for the breath-hold beam delivery. Subsequently, the patient was asked to breathe in slowly under fluoroscopy. Immediately after the marker position on the fluoroscopic image moved inside the tolerance range, the patient was asked to hold the breath and the VMAT beam was delivered. During the beam delivery, the breath-hold status was continuously monitored by checking if the deviation of the marker position exceeded the tolerance limit. As long as the marker stayed within the tolerance range, a segmented VMAT delivery continued for a preset period of 15 to 30 seconds depending on the breath-hold capability of each patient. As soon as each segmented delivery was completed, the beam interrupt button was pushed; subsequently, the patient was asked for free breathing. This procedure was repeated until all the segmented VMAT beams were delivered. A lung tumor case is reported here as an initial study. The proposed technique may be clinically advantageous for treating respiratory moving tumors including lung tumor, liver cancer, and other abdominal cancers.

## Introduction

Respiratory tumor movement needs to be managed during treatment. Many techniques have been proposed and passive breath-hold may be the simplest. However, the tumor position may not be accurately reproduced among planning computed tomography (CT) imaging, pre-treatment tumor localization and beam delivery; and therefore, additional margin may be required to consider the tumor position uncertainty.

To reduce the position uncertainty, spirometry was employed assuming good correlation between the tumor position and a lung volume [1]. However, Eccles et al. showed that the spirometry provided good intrafraction reproducibility of a diaphragm position but interfraction reproducibility was less satisfactory [2].

Meanwhile, a fluoroscopic image-guided approach was proposed by comparing the diaphragm position between digitally reconstructed radiography (DRR) and kilovolt (kV) fluoroscopic images during beam delivery, thereby assisting clinical staff to manually control the treatment beam during multiple breath-hold treatment [3]. In this technique, the planned diaphragm contour on the DRR image and the real-time fluoroscopic diaphragm image were compared for pre-determined gantry angles, facilitating static multi-field breath-hold radiotherapy. However, this technique may not be appliable to volumetric modulated arc therapy (VMAT) because real-time visual comparison between the planned diaphragm contour and the fluoroscopic diaphragm image was too labor intensive.

To solve this problem, a different fluoroscopic approach was proposed using implanted fiducial markers [4], where the diaphragm contour was replaced with contours of the fiducial markers. Although this technique worked well, a possible disadvantage may be that an unexpected intermediate beam interrupt due to a breath-hold failure during a segmented VMAT delivery cannot be well managed due to lack of the DRR for the failed gantry angle.

To avoid this possible problem, an advanced technique has been proposed, where a fluoroscopic marker image is continuously compared to a reference lateral line for monitoring breath-hold status during the entire VMAT delivery, the reference line being an aimed projected trajectory of the marker. In other words, the delivery can be restarted at any gantry angle after the marker position comes back to the reference lateral line. The purpose of this study is to describe the above procedure and demonstrate its clinical advantage for a respiratory moving tumor.

## Case presentation

**Figure 1:**
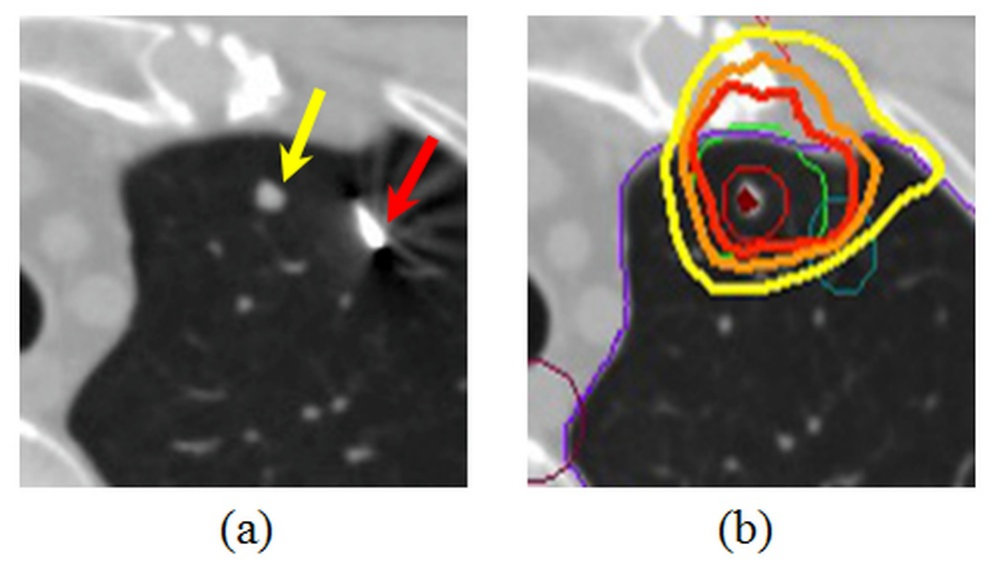
Computed tomography images (a) The yellow arrow shows a tumor whereas the red arrow is a fiducial marker. (b) The thick red, orange, and yellow lines are isodose contours of 3.0, 2.8, and 2.6 Gy/fraction, respectively. The thin red, green, and blue lines represent the clinical target volume, the planning target volume with an isotropic margin of 5 mm, and the marker regions, respectively.

We present a case of a 49-year-old male with a lung metastasis from hepatocellular carcinoma in the upper lobe of the left lung. He received radiotherapy to the lung metastasis according to the method described hereinafter. The proposed workflow started with acquiring planning CT images under deep inspiration breath-hold condition with a commercial gold coil marker, Visicoil 21G slim line (IBA Dosimetry, Schwarzenbruck, Germany) of diameter 0.5 mm and length 10 mm, implanted using CT guidance as close as possible to a tumor as shown in Figure 1a. Then the CT images were exported to a treatment planning system, Monaco (Elekta AB, Stockholm, Sweden). A single-arc coplanar VMAT plan (gantry rotation from 320° to 100° ) was created with an isotropic planning target volume (PTV) margin of 5 mm and a prescribed dose of 60 Gy in 20 fractions as indicated in Figure 1b. The plan was exported to a linac, Synergy (Elekta AB, Stockholm, Sweden), equipped with a kV fluoroscopic and cone-beam CT (CBCT) imager, Xray Volume Imaging (XVI).

Because VMAT beam-on-time typically exceeds 60 seconds, multiple breath-holds were required to complete the delivery. In other words, the single-arc VMAT beam was divided into several segmented VMAT beams each having different gantry start and stop angles. After performing CT imaging for the treatment planning, breath-hold training was given to each patient for optimizing the breath-hold and the following free breathing periods so that each segmented breath-hold VMAT delivery could be successfully completed.

In order to deliver segmented VMAT beams while the implanted marker stays at the planned breath-hold position, a DRR image at the gantry start angle was created in the Monaco TPS and transferred to the XVI. Subsequently, two lateral lines were drawn 2.5 mm above and below the center of the planned breath-hold marker position on the DRR image. Those lines were manually copied onto a fluoroscopic image window of the XVI display using a transparent sheet, each line being used as a tolerance limit for the breath-hold beam delivery.

Prior to the beam delivery, CBCT imaging under free-breathing condition was performed to adjust the position of the patient couch by matching bone anatomy between the planning CT and the CBCT images. Subsequently, the patient was asked to breathe in slowly under fluoroscopy. Immediately after the marker position on the fluoroscopic image moved inside the tolerance range, the patient was asked to hold the breath and the VMAT beam was delivered. During the beam delivery, the breath-hold status was continuously monitored by checking if the deviation of the marker position exceeded the tolerance limit. As long as the marker stayed within the tolerance range, a segmented VMAT delivery continued for a preset period of 15 to 30 seconds depending on the breath-hold capability of each patient. As soon as each segmented delivery was completed, the beam interrupt button was pushed; and then, the patient was asked for free breathing. This procedure was repeated until all the segmented VMAT beams were delivered. Even when an intermediate beam interrupt due to a breath-hold failure during each segmented beam delivery was observed, the remaining beam delivery can be safely performed by referring to the two lateral tolerance lines for reproducing the breath-hold status for any gantry angles. It was decided that patients unable to hold the breath at least for15 seconds were considered not applicable. The patient who could hold the breath for 20 seconds was selected for this study after written informed consent was obtained. In order to confirm that the marker position relative to the tumor remained unchanged, multiple breath-hold CBCT imaging was also performed with the marker being inside the tolerance limit, thereby allowing comparison of the marker positions between planning CT and the breath-hold CBCT images.

Video 1 shows a fluoroscopic movie showing the movement of the coil marker during the first coplanar segmented VMAT delivery (gantry rotation from 320° to 0° ) on the patient in reference to the two lateral lines (green color) giving a tolerance limit of 2.5 mm above and below the projected center of the marker. As long as the marker center stayed within the tolerance range, the segmented VMAT delivery continued for a preset period of about 25 seconds depending on the breath-hold capability of the patient. Unexpected intermediate beam interrupts due to a breath-hold failure during the segmented VMAT delivery can be well managed because the remaining beam delivery can be restarted at any gantry angle once the marker comes back within the tolerance range. In this lung tumor case, the total VMAT delivery time for a prescribed fraction dose of 3 Gy was approximately 115 sec with three beam interrupts and a 25 sec segmented beam delivery followed by 20 sec free breathing.

**Video 1:** Fluoroscopy A fluoroscopic movie showing the movement of the coil marker during the first coplanar segmented VMAT delivery (gantry rotation from 320° to 0°) on the patient in reference to the two lateral lines (green color) each giving a tolerance limit of 2.5 mm above and below the projected center of the marker.

## Discussion

Under the breath-hold condition, the marker moved toward the lateral direction while the gantry was rotating. The visibility of the marker varied depending on the gantry angle; however, it was possible for the present author to recognize the position during most of the entire beam delivery. If the marker was not visible due to the bone anatomy, the delivery was interrupted to optimize the brightness and the contrast of the XVI fluoroscopic image window. After confirming that the marker was located within the tolerance lines, the beam delivery was resumed.

As was described, a fluoroscopic marker image was continuously compared to the lateral tolerance lines for monitoring breath-hold status during the entire VMAT delivery. Consequently, the delivery could be restarted at any gantry angle once the marker position moved within the tolerance lines. This is a much safer technique compared to our previous report [4], where an unexpected intermediate beam interrupt due to a breath-hold failure during a segmented VMAT delivery had not been well managed due to lack of the DRR for the failed gantry angle. In addition, the present technique does not need the breathing monitoring by way of thoracoabdominal surface coordinates during VMAT delivery, which was required in our previous method [4].

It was reported that respiratory-gated VMAT resulted in four times longer treatment time when the breathing frequency was 15 cycles per minute compared to non-gated VMAT delivery [5]. The proposed segmented breath-hold technique may be much faster if the number of beam interrupts is relatively small. As mentioned earlier, in this lung tumor case, the total VMAT delivery time for a prescribed fraction dose of 3 Gy was approximately 115 sec with three beam interrupts and a 25 sec segmented beam delivery followed by 20 sec free breathing. The shorter delivery time may ensure more comfort to patients and thus better intrafraction position stability.

## Conclusions

An advanced multiple breath-hold segmented VMAT technique has been proposed under real-time fluoroscopic image guidance with implanted fiducial markers. The proposed technique may be clinically advantageous for treating respiratory moving tumors including lung tumors, pancreatic cancers, liver cancers, and other abdominal cancers.
